# Mechanical power of ventilation is associated with mortality in neurocritical patients: a cohort study

**DOI:** 10.1007/s10877-022-00805-5

**Published:** 2022-01-20

**Authors:** Xiaofeng Jiang, Yanhong Zhu, Shuai Zhen, Lei Wang

**Affiliations:** 1 Department of Anesthesiology, Jinhua Municipal Central Hospital, Jinhua, China; 2 Department of Anesthesiology, The First People’s Hospital of Pinghu, Jiaxing, China; 3 Department of Anesthesiology, The First People’s Hospital of Pinghu, 500 Sangang Road, Danghu Street, Pinghu, 314200 Zhejiang China

**Keywords:** Mechanical ventilation, Mechanical power, Mortality, Neurocritical care

## Abstract

This study aimed to determine the predictive relevance of mechanical power in the clinical outcomes (such as ICU mortality, hospital mortality, 90-day mortality, length of ICU stay, and number of ventilator-free days at day 28) of neurocritical patients. This is a retrospective cohort analysis of an open-access clinical database known as MIMIC–III. The study included patients who had sustained an acute brain injury and required invasive ventilation for at least 24 h. Demographic parameters, disease severity scores (Glasgow coma scale), comorbidities, vital signs, laboratory parameters and ventilator parameters were collected within the first 24 h of ICU admission. The main outcome was the relationship between MP and ICU mortality. A total of 529 patients were selected for the study. The critical value of MP was 12.16 J/min, with the area under the curve (AUC) of the MP was 0.678 (95% CI 0.637–0.718), and compared to the GCS scores, the MP performed significantly better in discrimination (DeLong’s test: *p* < 0.001). Among these patients elevated MP was associated to higher ICU mortality (OR 1.11; 95% CI 1.06–1.17; *p* < 0.001), enhanced the risk of hospital mortality, prolonged ICU stay, and decreased the number of ventilator-free days. In the subgroup analysis, high MP was associated with ICU mortality regardless of ARDS (OR 1.01, 95% CI 1.00–1.02, *p* = 0.009; OR 1.01, 95% CI 1.00–1.02, *p* = 0.018, respectively) or obesity (OR 1.01, 95% CI 1.00–1.02, *p* = 0.012; OR 1.01, 95% CI 1.01–1.02, *p* < 0.001, respectively). In neurocritical care patients undergoing invasive ventilation, elevated MP is linked to higher ICU mortality and a variety of other clinical outcomes.

**Supplementary Information:**

The online version contains supplementary material available at 10.1007/s10877-022-00805-5.

## Introduction

There are different conditions for admission to neurocritical care (NCC). The most common reason is acute brain injury, which includes traumatic brain injury, subarachnoid hemorrhage, and intracranial hemorrhage [1]. The altered level of sensorium of these individuals necessitates intubation and invasive mechanical ventilation to prevent aspiration and life-threatening consequences, such as hypoxia and hypercapnia [2]. Moreover, patients are also at a high risk of respiratory complications and mortality during their stay in the NCC [3, 4]. The protective lung ventilation (PLV) strategy can reduce ventilator-induced lung injury (VILI) and improve the prognosis of patients with invasive ventilation [5, 6]. However, the PLV strategy is controversial, and is contraindicated for NCC patients because the use of permissible hypercapnia and increased airway pressure can increase the intracranial pressure (ICP) during recruitment maneuvers [7, 8]. Therefore, a new ventilator parameter is needed to guide ventilation and to reduce the adverse outcomes from VILI and complex brain-lung interactions.

The mechanical power of ventilation (MP) refers to the amount of energy transmitted from the ventilator to the airway and lungs per unit of time [9]. It is determined by peak inspiratory pressure, plateau pressure, tidal volume, positive end-expiratory pressure (PEEP), and respiratory rate [9, 10]. Therefore, it can be presumed that the MP is better than any individual ventilator parameter in evaluating the prevalence of respiratory complications and mortality [9, 10]. Recent studies have demonstrated that the mortality of invasively-ventilated ICU patients is independently associated with MP [11, 12]. However, there are only few investigations that have determined the connection between MP and outcomes in neurocritical patients. We hypothesize that elevated MP can be used as one of the modalities to predict mortality in NCC patients on mechanical ventilatory support.

Therefore, this study aimed to determine the predictive relevance of mechanical power in the clinical outcomes of neurocritical patients.

## Materials and methods

### Data source

An open-access clinical database known as the Multiparameter– Intelligent Monitoring in Intensive Care III (MIMIC III) was used to conduct this retrospective cohort analysis [13]. The database has medical records of approximately 50,000 ICU patients admitted from 2001 to 2012 to the Beth Israel Deaconess Medical Center (BIDMC, Boston, MA, USA). The National Institutes of Health's course “Protecting Human Research Participants” is required to access the database. The study was conducted in accordance with the Declaration of Helsinki. Both BIDMC and the Massachusetts Institute of Technology (MIT) Institutional Review Boards agreed to approve this project (certification number: 9322422), and informed consent was waived for this retrospective study.

### Participant selection criteria

Patients who had sustained an acute brain injury and required invasive ventilation for at least 24 h were selected. The exclusion criteria were as follows: missing > 5% data, extubation or death within the first 24 h, received pressure support ventilation, and/or missing ventilation variables associated with MP.

### Data collection

For data extraction, the PostgreSQL tool was utilized. These variables were collected or calculated: demographic parameters, disease severity scores (Glasgow coma scale [GCS] [14]), comorbidities, vital signs, laboratory parameters, ventilator parameters. Mechanical power (MP) was derived using the following equation [10, 11]: MP(J/min) = 0.098 × Vt × RR × (PIP−ΔP × 0.5), where the driving pressure (ΔP) = PIP−PEEP [15] denotes the quantity of energy generated, and released into the airway and the lungs by mechanical ventilation. The mean values of the vital signs, laboratory measures, and MP within the first 24 h were used due to repeated measurements of these patients. Variables were collected for well relaxed patients (maintained deeply sedated or paralyzed). Identify spontaneous breathing by comparing RRset and RRmeasured, and RRmeasured being higher than RRset was regarded as evidence of spontaneous breathing. The MP during the first day of ventilation was selected because, in patients with traumatic brain injury, ventilator settings should be modified to more “lung-protective” values during the first 24 h [16].

The primary outcome was ICU mortality. Secondary outcomes were hospital mortality, 90-day mortality, length of ICU stay (ICU_LOS), and number of ventilator-free days at day 28 (VFD_28, defined as the number of days between effective weaning and day 28). Individuals who died prior to weaning were considered to have no ventilator-free days.

### Statistical methods

Patients were grouped according to ICU mortality. The median and interquartile ranges of continuous variables are displayed. Percentages were used to present the categorical variables. The Mann–Whitney *U* test was used to compare continuous data while the chi-square or Fisher's exact test was used to compare categorical data.

The predictive validity of the MP and GCS scores were determined using the area under the receiver operating characteristic curve (AUROC). Delong’s method was applied to test the AUROC difference.

The correlation between MP and the clinical findings of patients with brain injury was evaluated using univariable (non-adjusted) and multivariable (adjusted) regression. Relevant factors influencing the results including age, sex, ethnicity, BMI, admission reason, admission type, comorbidities, GCS, heart rate, MAP, SpO_2_, temperature, pH, PaO_2_/FiO_2_, and PaCO_2_ were incorporated into the model. These variables were chosen based on their clinical relevance and use. The final models were constructed using a stepwise backward elimination method with a significance level of 0.05. The impact of the driving pressure has recently been questioned in patients with obesity [17]. Thus, for obese individuals with a BMI more than 30 kg/m^2^, a subgroup analysis was performed. Further subgroup analysis was also performed to determine whether MP affected ICU mortality based on the presence of ARDS.

The statistical analysis was achieved with the help of SPSS version 22.0 (IBM, Armonk, NY, USA). A two-sided *p* value of less than 0.05 was used to determine statistical significance.

## Results

**Fig. 1:**
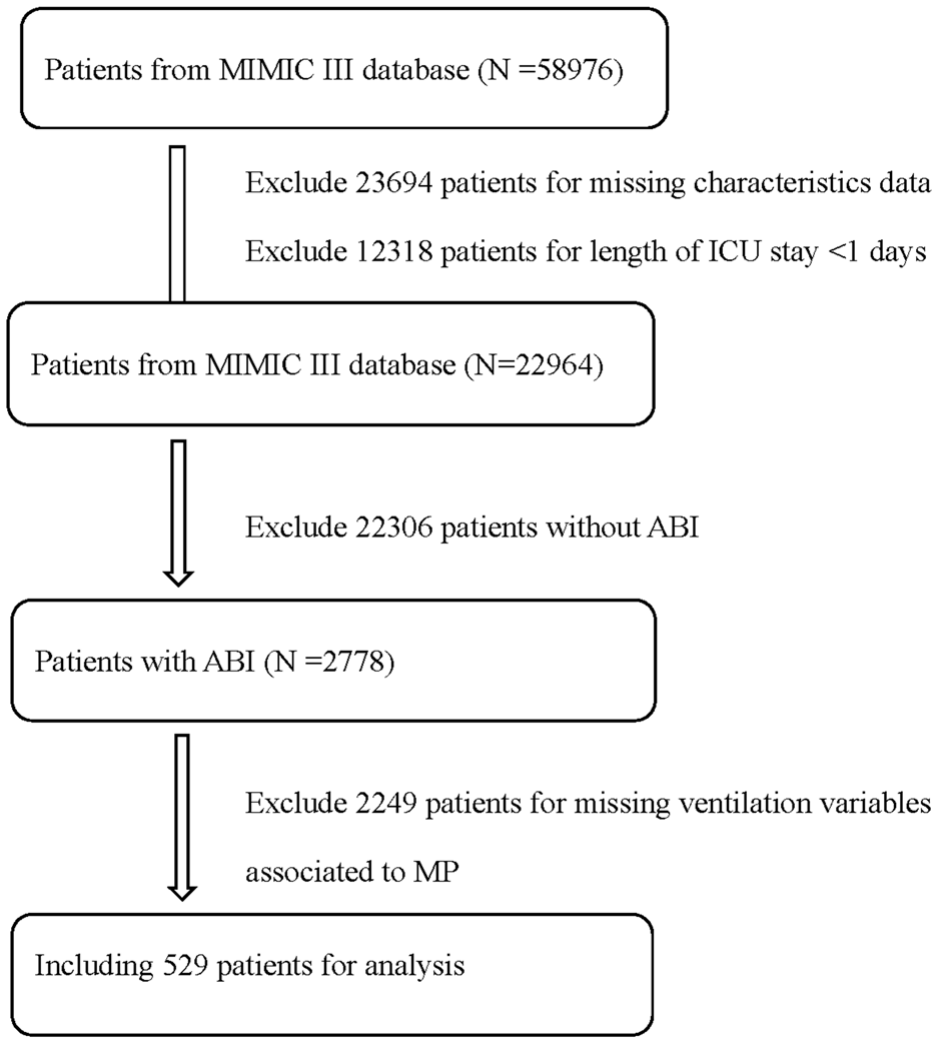
The process of participation selection and exclusion

A total of 529 patients were selected for the study (Fig. 1). The baseline characteristics of the participants who survived or died during their ICU stay are shown in Table 1. Compared with survivors, non-survivors tend to be older, with higher prevalence of hypertension and renal failure, lower body temperature, PaO_2_/FiO_2_ and PaCO_2_ values, higher PEEP, PIP, respiratory rate, and higher minute ventilation. Additionally, MP was significantly lower for survivors (10.3 [8.3–13.2]) than non-survivors (13.4 [10.1–17.6]) (*p* < 0.001).

**Table 1:** Comparisons of demographics between survivors and non–survivors

Baseline characteristics	Survivors (*n* = 394)	Non-survivors (*n* = 135)	*p* value
Age, years	58.1 (41.9–73.0)	64.0 (50.7–79.6)	0.006
Male gender	239/394 (60.7)	77/135 (57.0)	0.459
Weight, kg	76.5 (68.0–87.7)	76.0 (64.0–90.0)	0.616
Height, cm	171 (165–178)	171 (163–178)	0.686
BMI, kg/m^2^	25.9 (23.4–29.6)	26.0 (22.1–30.5)	0.701
PBW, kg	66.2 (57.1–73.1)	66.9 (54.7–73.1)	0.611
Admission type			0.071
Selective	14/394 (3.6)	0/135 (0)	
Emergency	379/394 (96.2)	135/365 (100)	
Urgent	1/394 (0.2)	0/135 (0)	
Admission reason			
SDH	26/394 (6.6)	11/135 (8.1)	0.004
SAH	46/394 (11.7)	29/135 (21.5)	
ICH	112/394 (28.4)	45/135 (33.3)	
Trauma	210/394 (53.3)	50/135 (37.1)	
Ethnicity			
White	268/394 (68.0)	71/135 (52.6)	0.004
Black	21/394 (5.3)	8/135 (5.9)	
Other	105/394 (26.6)	56/135 (41.5)	
Comorbidities			
CHF	30/394 (7.6)	13/135 (9.6)	0.460
Cardiac arrhythmias	70/394 (17.8)	30/135 (22.2)	0.254
Valvular disease	16/394 (4.1)	8/135 (5.9)	0.369
Hypertension	16/394 (4.1)	19/135 (14.1)	< 0.001
Diabetes	77/394 (19.5)	23/135 (17.0)	0.521
Chronic pulmonary condition	43/394 (10.9)	16/135 (11.9)	0.765
Renal failure	17/394 (4.3)	15/135 (11.1)	0.004
Liver condition	21/394 (5.3)	13/135 (9.6)	0.079
ARDS			
None	253/394 (64.2)	73/135 (54.1)	0.045
Mild	83/394 (21.1)	28/135 (20.7)	
Moderate	42/394 (10.6)	25/135 (18.5)	
Severe	16/394 (4.1)	9/135 (6.7)	
Severity of illness			
GCS scores	14 (10–15)	15 (6–15)	0.481
Vital signs in the beginning of ventilation			
Heart rate, bpm	85 (75–96)	84 (73–96)	0.504
MAP, mmHg	82 (76–88)	82 (77–88)	0.837
SpO_2_, %	99 (98–100)	99 (97–100)	0.145
Temperature, °C	37.2 (36.8–37.6)	36.9 (36.4–37.4)	< 0.001
Laboratory in the beginning of ventilation			
p H	7.40 (7.34–7.44)	7.41 (7.33–7.46)	0.383
PaO_2_/FiO_2_, mmHg	356 (254–465)	312 (200–424)	0.007
PaCO_2_, mmHg	40 (35–45)	36 (31–42)	< 0.001
FiO_2_	50 (40–70)	50 (50–100)	0.011
First day of ventilation parameters			
Tidal volume, ml/kg PBW	7.8 (6.9–8.7)	7.9 (7.0–8.8)	0.515
PEEP, cmH_2_O	5 (5–5)	5 (5–8)	< 0.001
PIP, cmH_2_O	18 (16–22)	22 (19–26)	< 0.001
Respiratory rate, bpm	18 (16–20)	20 (17–22)	< 0.001
Minute ventilation, L/min	8.8 (7.8–10.2)	10.0 (8.1–12.3)	< 0.001
MP, J/min	10.3 (8.3–13.2)	13.4 (10.1–17.6)	< 0.001

Data are median (interquartile range) or No/Total (%)

*BMI* body mass index; *PBW* predicted body weight, *SDH* subdural hemorrhage, *SAH* subarachnoid hemorrhage, *ICH* intracranial hemorrhage, *CHF* congestive heart failure; *ARDS* acute respiratory distress syndrome, *GCS* Glasgow coma scale, *bpm* beats per minute, *SpO*_*2*_ pulse oximetry, *MAP* mean arterial blood pressure,; *FiO*_*2*_ inspired fraction of oxygen, *PEEP* positive end-expiratory pressure, *PIP* peak inspiratory pressure, *MP* mechanical power

MP(J/min) = 0.098 × Vt × RR × (PIP − ΔP × 0.5); ΔP = PIP − PEEP

The predictive validity of the MP and GCS scores is presented in Table 2. The critical value of MP was 12.16 J/min, with an AUC of 0.678 (95% CI 0.637–0.718, *p* < 0.001), corresponding to a 63.7% predictive sensitivity and a 68.3% predictive specificity. And compared to the GCS scores, the MP performed significantly better in discrimination (DeLong’s test: *p* < 0.001). When compared to MP, the GCS-based MP did not show improvement in discrimination (DeLong’s test: *p* = 0.431).

**Table 2:** Predictive validity for the MP and the GCS scores

Parameters	AUROC	Lower limit of 95% CI	Upper limit of 95% CI
GCS scores	0.519	0.475	0.562
MP (J/min)	0.678	0.637	0.718
MP + GCS	0.687	0.646	0.726

The critical value of MP was 12.16 J/min, with an AUC of 0.678 (95% CI 0.637–0.718, *p* < 0.001), corresponding to a 63.7% predictive sensitivity and a 68.3% predictive specificity

The discrimination of MP was signifcantly better than the GCS scores (*p* < 0.001 for DeLong’s test). The MP based on GCS scores was not able to improve the discrimination as compared to the MP (*p* = 0.431 for DeLong’s test)

*GCS* glasgow coma scale, *MP* mechanical power, *CI* confdence interval

**Fig. 2:**
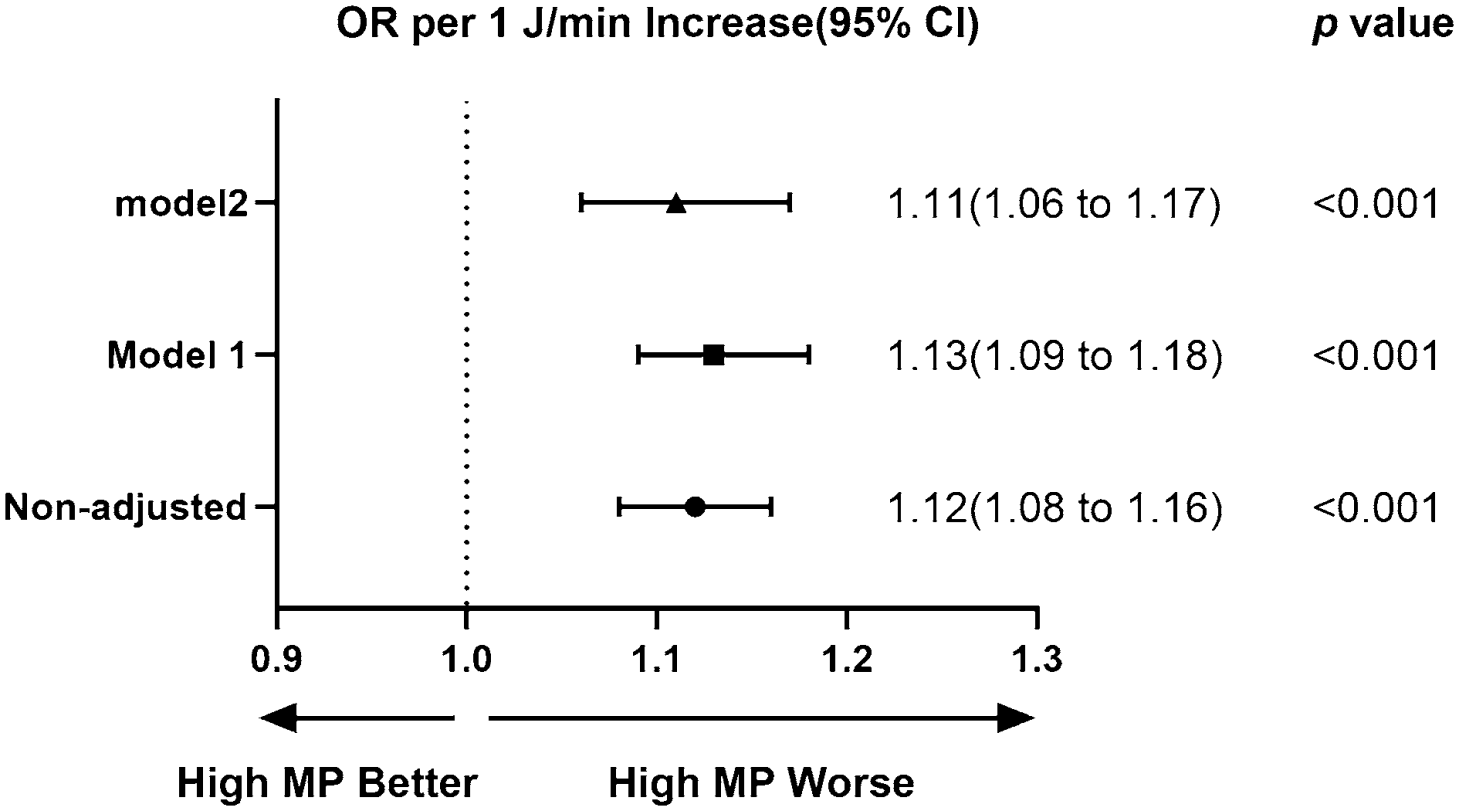
MP in the first 24 h of ventilation and ICU mortality. Model 1 was adjusted for the confounders age, sex and ethnicity and BMI. Model 2 was adjusted for the confounders, including age, sex, ethnicity, BMI, admission reason, admission type, comorbidities, GCS, heart rate, MAP, SpO_2_, temperature, pH, PaO_2_/FiO_2_, PaCO_2_. The odds ratio represents the odds of death per 1 J/min increase in MP. MP: mechanical power

**Fig. 3:**
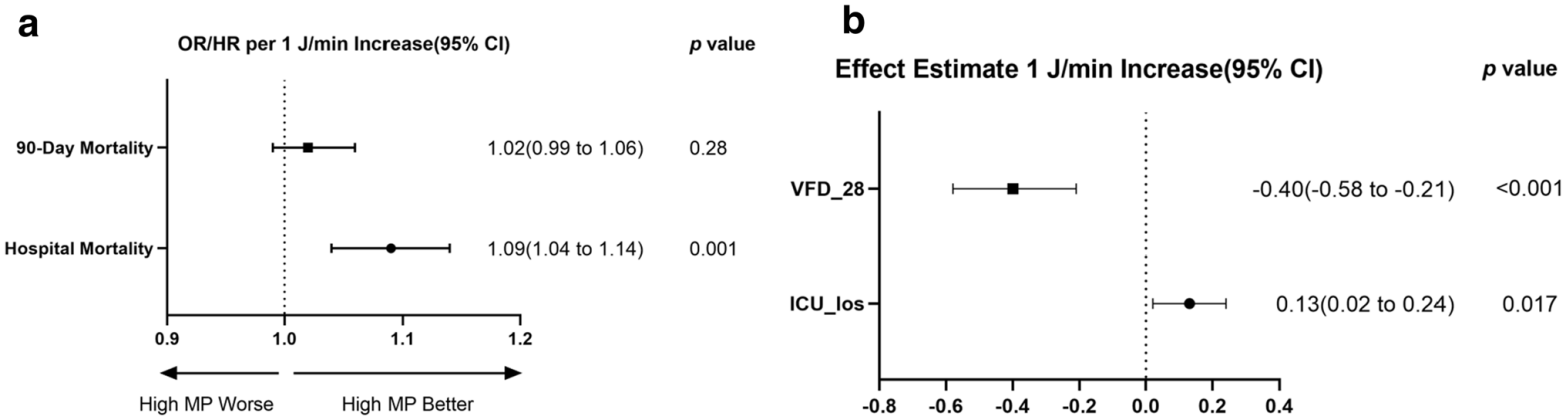
MP in the first day of ventilation and secondary outcomes. **a** Odds ratio represents the odds of death per 1 J/min increase in MP. **b** Effect estimates and 95% confidence interval from the multivariable linear regression for VFD_28 and ICU_los. Effect estimate refers to the change in the outcome variable per 1 J/min increase in MP. MP: mechanical power; VFD_28: Ventilator-free days at day 28; *ICU* intensive care unit, *LOS* length of stay

Univariate and multivariate logistic regression models (Fig. 2) showed that MP was associated to elevated ICU mortality risk (adjusted OR 1.11; 95% CI 1.06–1.17; *p* < 0.001). Meanwhile, multivariate analyses were also performed for the other ventilation parameters. PEEP, PIP and MV were also associated with ICU mortality (see Additional file 1: Table S1). Figure 3 depicts the results of the secondary outcome multivariate analysis. Elevated MP dramatically enhanced the risk of hospital mortality but not 90-day mortality (Fig. 3a), prolonged ICU stay, and decreased the number of ventilator-free days (Fig. 3b).

**Fig. 4:**
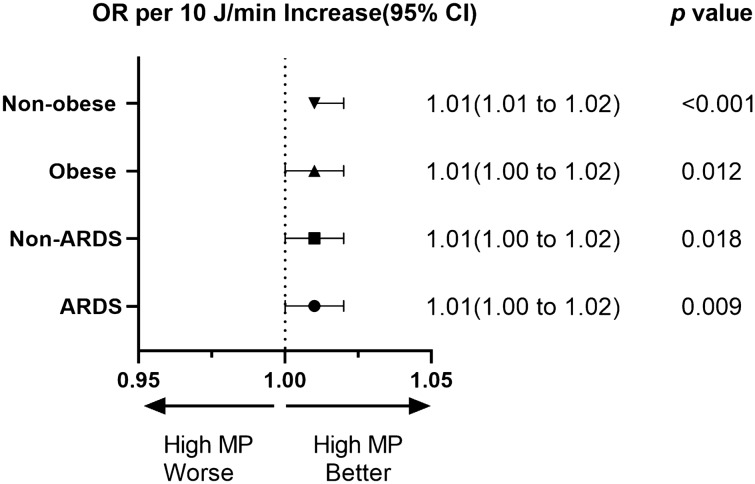
Subgroup analysis of the association between MP and ICU mortality according to presence of ARDS, obesity or not. The odds ratio represents the odds of death per 10 J/min increase in MP. *MP* mechanical power, *ARDS* acute respiratory distress syndrome

Figure 4 shows that high MP was associated with ICU mortality regardless of ARDS (OR 1.01, 95% CI 1.00–1.02, *p* = 0.009; OR 1.01, 95% CI 1.00–1.02, *p* = 0.018, respectively) or obesity (OR 1.01, 95% CI 1.00–1.02, *p* = 0.012; OR 1.01, 95% CI 1.01–1.02, *p* < 0.001, respectively).

## Discussion

**Fig. 5:**
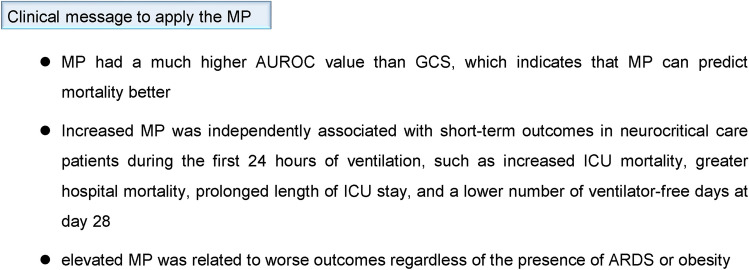
Clinical message to apply the MP

This study found that: (a) MP had a much higher AUROC value than GCS, which indicates that MP can predict mortality better; (b) MP was independently associated with increased ICU mortality in neurocritical care patients during the first 24 h of ventilation; (c) increased MP was independently related to greater hospital mortality, prolonged length of ICU stay, as well as a lower number of ventilator-free days at day 28 and (d) elevated MP was related to worse outcomes regardless of the presence of ARDS or obesity (Fig. 5).

Patients with and without ARDS benefit from a protective lung ventilation (PLV) strategy that includes low tidal volume, recruitment maneuvers, and PEEP [18]. However, PLV is rarely employed in NCC patients because its potential to alter important parameters such as PaO_2_, PaCO_2_, and CBF [19]. Low tidal volume might result in a rise in PaCO_2_, which could lead to cerebral vasodilation and subsequently, an increase in ICP [20]. Previous investigations have demonstrated that apart from hypercapnia, hyperventilation may also be harmful in NCC patients, as it can lead to serious hypocapnia and resultant brain tissue hypoxia (BTH) and cerebral compliance and blood flow velocity might be compromised [21, 22]. Recruitment maneuvers and PEEP are both beneficial strategies for improving oxygenation, and optimization of ventilation-perfusion failure by lowering alveolar end-expiratory collapse and opening collapsed alveoli [23, 24]. However, PEEP and recruitment maneuvers can increase CVP and intrathoracic pressure, and decrease right atrial return. All of these may contribute to elevations in ICP in patients with cerebral disorders [25].

MP is composed of all aspects of mechanical ventilation, in contrast to the typical variables of the PLV strategy. In theory, MP also integrates the mechanical damage and the impact on brain physiology by ventilator settings. Therefore, it can be presumed that MP can best predict clinical results. Zhang et al. [12] found that MP had somewhat higher discrimination in predicting mortality than any other individual variable. However, the findings of this study were not tested through direct comparisons of MP and GCS scores. GCS is commonly utilized by clinicians in making treatment decisions, as well as in estimating disease outcomes [26, 27]. This study proved that MP had superior discrimination ability than GCS in predicting mortality. Furthermore, this finding also confirms that GCS can be affected by various factors such as ventilator settings that can negatively impact the accuracy and discrimination of the prediction model [28].

In addition, MP was examined in previous studies for its relationship with mortality, and was found to be a strong predictor of mortality [11, 12]. However, the effects of MP on neurological patients were not demonstrated in these two investigations. Our findings show that MP also has prognostic capability in neurological patients with controversial ventilator conditions. In the future, caregivers may set the ventilator parameters of NCC patients based on the MP to further improve the prognosis by preventing VILI and to reduce the impact of ventilator settings on brain physiology.

There are several limitations to this study. First, ventilator variables in the first 24 h hours were used to predict mortality. In fact, the patient's survival prospects might be dramatically influenced by the ventilator settings that follow. However, the ventilator variables within the first 24 h can reflect clinical practice more effectively. Further research into the temporal variations of ventilator settings may be beneficial. Second, this study did not report about pneumonia, atresia, or barotrauma related to the ventilator because they were not consistently present in the examined databases. Third, it is probable that the airway pressure was not acquired under continuous standard circumstances because the datasets used in this analysis were derived from publicly available sources. Finally, due to the MIMIC-III's lack of prone positioning, the results may be confounded. Although we have found that high MPs are linked to mortality, more evidence from further studies is needed.

## Conclusions

In neurocritical care patients undergoing invasive ventilation, elevated MP is associated with higher ICU mortality and a variety of other clinical outcomes. Due to the ease of determining MP using ventilator parameters, it can be used as a modality for the prediction of mortality in NCC patients on mechanical ventilatory support.

## Supplementary Information

Below is the link to the electronic supplementary material.

Supplementary file1 (DOCX 13 kb)

## Data Availability

The datasets of the current study are available from the corresponding author on reasonable request.
