# Supplementary material for: Mechanical power of ventilation is associated with mortality in neurocritical patients: a cohort study
Source: J Clin Monit Comput. 2022 Jan 20;36(6):1621–8. doi: 10.1007/s10877-022-00805-5 (PMC9637601; doi:10.1007/s10877-022-00805-5)
Supplement: Supplementary file 1 — Supplementary file1 (DOCX 13 kb) [file 10877_2022_805_MOESM1_ESM.docx]

STable 1. Predictive performance of ventilatory variables for ICU mortality

| Parameters | OR | 95% CI | p |
| --- | --- | --- | --- |
| Tidal volume, ml/kg PBW | 1.04 | 0.89–1.20 | 0.626 |
| PEEP, cmH2O | 1.16 | 1.02–1.31 | 0.02 |
| PIP, cmH2O | 1.15 | 1.09–1.20 | <0.001 |
| Minute ventilation, L/min | 1.22 | 1.08–1.38 | 0.001 |
| MP, J/min | 1.11 | 1.06-1.17 | <0.001 |
| Odds ratios (OR) and 95% confidence intervals (CI) of ICU mortality calculated with multivariable (adjusted) regression  PBW: predicted body weight; PEEP: positive end-expiratory pressure; PIP: peak inspiratory pressure; MP: mechanical powe | | | |
